# Spatiotemporal evolution and clustering patterns of settlements from the Neolithic to the Bronze Age (9000–3000 BP) in the Songshan Mountain region, China

**DOI:** 10.1371/journal.pone.0351644

**Published:** 2026-07-22

**Authors:** Lijie Yan, Xia Wang, Li Zhang, Fei Teng

**Affiliations:** Institute of Geography, Henan Academy of Sciences, Zhengzhou, China; Maria Curie-Sklodowska University: Uniwersytet Marii Curie-Sklodowskiej, POLAND

## Abstract

This study proposes a transparent and methodologically explicit GIS-based framework for delineating prehistoric settlement clusters and tracking their spatiotemporal evolution. Integrating nearest-neighbor analysis, kernel density estimation, least-cost path (LCP) modeling, and K-medoids clustering, we analyze 1,975 settlement sites from four successive cultural phases (Peiligang, Yangshao, Longshan, and Xia-Shang; 9000–3000 BP) in the Songshan Mountain region, a core area of early Chinese civilization. Results show persistent clustered distribution across all periods, with aggregation intensity peaking during the Xia-Shang period. High-density zones shifted progressively westward, and by the Xia-Shang period, a spatial pattern that appears consistent with a potential dual-core structure can be identified centered on Yanshi and Zhengzhou. K-medoids clustering reveals a marked reduction in optimal cluster numbers from nine to two despite a five-fold increase in site numbers. This trend is consistent with spatial integration and is suggestive of socio-political centralization. Environmental factors provided the primary context for settlement distribution from the Peiligang to Longshan period, but spatial patterns suggest socio-political factors may have exerted an important influence on aggregation during the Xia-Shang period even under deteriorating climate conditions. Our framework offers a replicable approach for analyzing settlement clustering and human-environment interactions.

## 1. Introduction

Settlements, as fundamental units supporting human production, life and social activities, serve as key markers of the progression of human civilization [1]. Their location, morphology and spatial layout reflect the integrated influence of natural conditions and socio-environmental factors, vividly illustrating the dynamic interaction between humans and their environment within specific geographic contexts [2]. In recent years, the mechanisms underlying settlement-environment interactions during prehistory have become a focal point of interdisciplinary research [3–6], with growing attention to quantitative analysis of macro-scale settlement aggregation patterns and their spatiotemporal dynamics.

Rapid advances in spatial information technologies—particularly Geographic Information Systems (GIS) and Remote Sensing (RS)—have driven methodological innovations in archaeology, enabling detailed analysis of settlement patterns. A substantial body of research has focused on documenting the relationship between individual site locations and environmental variables. For instance, Wienhold employed GIS to analyze topography at multiple scales, revealing links between prehistoric sites and hydrological features in central Arizona [7]. At a continental scale, Wagner et al. mapped the spatial-temporal distribution of over 36,000 sites in northern China, documenting broad shifts in settlement concentrations from the Weihe and Yellow River basins to eastern China [8]. Rajani utilized RS and GIS to identify archaeological features in India, assessing the impacts of land-use change on site preservation [9]. More recently, Sulaiman et al. analyzed the spatial distribution of archaeological sites across Saudi Arabia, demonstrating the continued relevance of site-level distribution mapping [10]. These studies have established the foundational value of GIS for understanding site-environment relationships but largely treat settlements as discrete points rather than as components of larger spatial aggregations.

A complementary line of inquiry has examined settlements as organized systems, focusing on regional patterns and hierarchical structures. Li et al. used GIS to examine scale-level patterns of Neolithic–Bronze Age settlements in the Gansu-Qinghai region, revealing how settlements of different sizes were distributed and how social organization evolved [11]. In the Northern Haidai region, Zou et al. analyzed the spatiotemporal patterns of settlements and their driving mechanisms, elucidating the roles of the natural environment, social organization, and subsistence strategies [12]. Focusing on the Songshan Mountain area—the core of our study, Tian et al. assessed the influence ranges of urban sites from the Neolithic to Bronze Age, implicitly recognizing that settlements functioned within networks of interaction and competition [13]. Yan et al. explored the relationship between prehistoric settlement location and rivers in the same region, highlighting the persistent importance of water resources [14]. Furthermore, a series of geoarchaeological studies by Lu et al. in the Songshan Mountain area demonstrated how Holocene alluvial landscape evolution and prolonged landscape stability sustained the continuous development of ancient civilizations, providing crucial environmental context for understanding settlement patterns [15–17]. While these studies have advanced our understanding of inter-settlement relationships and environmental settings, they have not explicitly delineated or analyzed settlement clusters as coherent spatial units whose boundaries and internal dynamics could be tracked through time.

Explicit applications of cluster analysis to prehistoric settlement data, while still evolving, have demonstrated the potential of this approach for understanding spatial organization. Within the Zhengzhou-Luoyang area—the core of our study region.Bi et al. employed the DBSCAN (density-based spatial clustering of applications with noise) algorithm to analyze Neolithic settlement sites across four cultural periods, revealing that the main settlement cluster shifted from the southern Songshan Mountain area to the Yi-Luo River Basin and persisted there through time [18]. Similarly, Zhang et al. developed a PATHCLUST clustering method specifically tailored to the complex terrain of the Zhengzhou area, identifying four major settlement clusters (Yi-Luo, Jialu, Ying, and Shuangji River Basins) during the Yangshao period and demonstrating that sites near cluster centers with larger areas exhibited potential as regional centers [19]. In the Linfen area of the Fenhe River Basin, Liu et al. integrated cluster analysis with climatic and topographic factors to characterize settlement evolution, demonstrating how terrain constraints shaped aggregation patterns by employing resistance distance rather than Euclidean distance to better simulate actual movement costs [20]. Beyond these regional applications, the archaeological community has increasingly recognized the importance of method evaluation and diversification. Diaz-Rodriguez et al. systematically evaluated the effectiveness of K-means, DBSCAN, and percolation analysis on Palaeolithic site distributions in Galicia, Spain, providing critical insights into the strengths and limitations of each method for archaeological problems at regional scales and demonstrating that percolation analysis and DBSCAN outperformed K-means for identifying clustered distributions [21]. Approaches like Ripley’s K function have enabled researchers such as Harrower et al. to detect spatial patterns among settlements across multiple scales, offering a means to distinguish between environmental and social drivers of aggregation [22].

Despite these valuable contributions, several gaps remain. First, while clustering studies exist for the Zhengzhou-Luoyang area, they predominantly focus on individual periods (e.g., Yangshao) or employ methods (e.g., DBSCAN) that, while effective for density-based clustering, do not incorporate movement costs reflecting the actual connectivity constraints of prehistoric human activities. As comparative methodological studies have shown [21], the choice of clustering algorithm and its underlying distance metric significantly impacts the resulting cluster configurations. Second, few studies have systematically tracked cluster evolution across multiple periods within a consistent, replicable framework that allows for direct cross-period comparison. Third, the shifting balance between environmental and socio-political drivers in shaping the emergence, persistence, and transformation of settlement clusters—particularly in core areas of early Chinese civilization like the Songshan Mountain region—remains underexplored.

To address these gaps, this study aims to answer two key questions: (1) how did settlement clusters in the Songshan Mountain region evolve spatially and temporally from the Neolithic to the Bronze Age? (2) What were the respective roles of regional environmental change and social complexification in driving these patterns, and did their relative importance shift over time? We therefore propose a comprehensive framework that (1) uses nearest-neighbor analysis to quantify agglomeration, (2) employs kernel density estimation to characterize spatial distribution, (3) applies Least-cost path modeling to delineate clusters and track their spatial-temporal evolution, and (4) investigates the feedbacks between cluster dynamics, environmental change and societal development as well as the coupling relationship with prehistoric livelihood economy. Applied to the Songshan Mountain region, which is the core area of early Chinese civilization, this approach demonstrates how millennial-scale climate oscillations and human activities jointly regulated settlement nucleation and dispersal. Our findings enhance understanding of civilization origins and social complexity in the Songshan Mountain vicinity and offer a methodologically transparent and replicable in principle framework for delineating settlement clusters in other regions.

## 2. Study area

**Fig 1:**
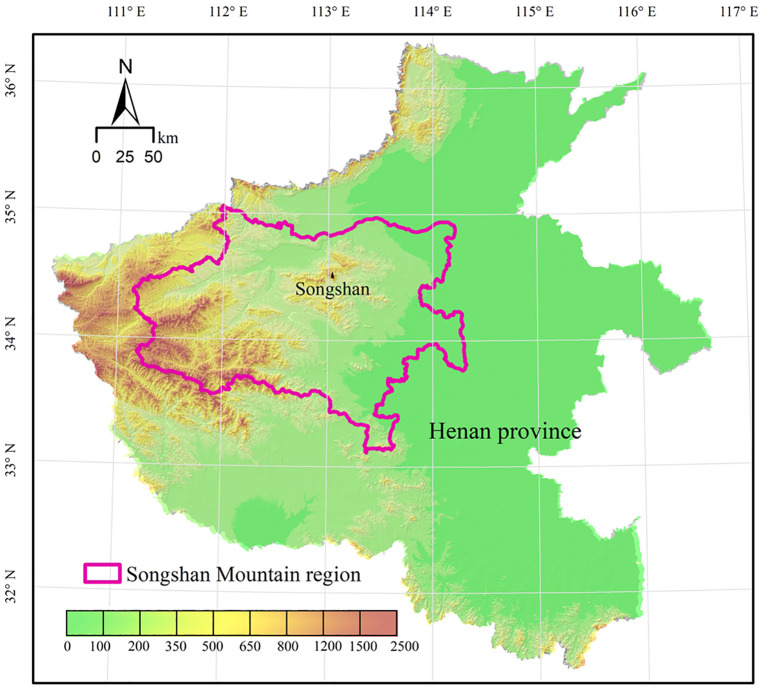
The geographic location of the study area. This figure was created by the authors in ArcGIS 10.8. Digital Elevation Model (DEM) data were obtained from the Geospatial Data Cloud site, Computer Network Information Center, Chinese Academy of Sciences (http://www.gscloud.cn). Administrative boundaries were derived from OpenStreetMap (https://www.openstreetmap.org), © OpenStreetMap contributors, available under the Open Database License (ODbL).

The Songshan Mountain region, centered on Songshan Mountain, encompasses the municipalities of Zhengzhou, Luoyang, Xuchang and Pingdingshan. It lies between longitudes 111°8’20”E to 114°19’20”E and latitudes 33°6’50”N to 35°3’30”N, spanning approximately 294 km east-west and 214 km north–south, with a total area of about 35,600 km^2^ (Fig 1). An extensive river network, dominated by the Yellow River system in the north and the Huaihe River system in the south, provides abundant water resources that facilitated early agricultural development and underpinned the emergence and growth of ancient civilizations [23].

Characterized by a high concentration of cultural sites in the middle and lower reaches of the Yellow River, the Songshan Mountain region has long been a focal point for archaeological, origins-of-civilization, environmental-archaeological and settlement-archaeological research [15–17]. From the Neolithic through the Xia-Shang dynastic period, four successive cultural phases are recognized here: Peiligang period (9000–7500 BP), Yangshao period (7500–5000 BP), Longshan period (5000–4000 BP) and Xia-Shang period (4000–3000 BP). Research on the division of settlement clusters across these phases can provide a more profound understanding of prehistoric social organization. This study is therefore critical for reconstructing the spatial distribution of settlements during prehistory and elucidating the origins of Chinese civilization.

## 3. Data and methods

### 3.1. Data

Digital Elevation Model (DEM) data were obtained from the Data Sharing Platform of the Institute of Geographic Sciences and Natural Resources Research, Chinese Academy of Sciences (http://www.gscloud.cn/), with a spatial resolution of 30 m × 30 m. Slope was derived from the DEM using standard terrain analysis methods. The hydrological network of the study area was generated through the GIS hydrological analysis module by extracting stream channels and catchments from the DEM.

Settlement site data were entirely obtained from the Third National Cultural Relics Survey of China, which provides standardized and accurate geographic coordinates for prehistoric sites in the region. A total of 1,975 sites were included, covering four cultural phases: 118 Peiligang-period sites, 563 Yangshao-period sites, 660 Longshan-period sites, and 634 Xia-Shang-period sites. Of these, 312 sites (15.8%) were field-checked using handheld GPS (error ±5–10 m). The remaining 1,663 sites (84.2%) are map-based; estimated positional error ≤50 m based on map scale and georeferencing validation. This accuracy is sufficient for macro-regional analysis (grid resolution 30 m, cluster spacing 10–50 km). A spatio-temporal database of settlement sites was established, incorporating key attributes such as chronological phase, site area (in 10,000 m²), cultural layer thickness (in cm), and representative unearthed artifacts.

After excluding sites that fell outside the continuous cost surface or lacked valid coordinates for least-cost path calculation, the effective sample sizes for clustering analysis were 117 for Peiligang, 561 for Yangshao, 659 for Longshan, and 632 for Xia-Shang (see S3 File for the exact site lists). The nearest-neighbor and kernel density analyses (Sections 4.1–4.2) used the full dataset of 1,975 sites.

Paleoclimate data were integrated from regionally representative proxy indicators: (1) regional and global temperature reconstructions based on multiple proxy records [24]; (2) annual precipitation estimates derived from pollen assemblages in Gonghai Lake (35°42′N, 112°18′E) [25];(3) moisture and vegetation indicators from the Xingyang Basin based on n-alkane indices (C₂₇ ₊ ₂₉/C₃₁ ₊ ₃₃ ratio and ACL, Average Chain Length), reflecting local hydrological conditions and vegetation succession [26], (4) pollen-based vegetation and humidity indicators from the Mianchi Basin [27], which provide supplementary evidence for regional vegetation dynamics and moisture changes, enhancing the consistency and robustness of paleoclimate interpretation.

### 3.2. Methods

#### 3.2.1. Methodology for assessing the aggregation degree of settlements.

The nearest neighbor distance method was employed to confirm the presence of clustering [28,29]. The Nearest Neighbor Index (NNI) was calculated as the ratio of observed average nearest-neighbor distance to expected average distance under random distribution:


$$\begin{array}{c} NNI=\frac{D(NN)}{D(ran)} \end{array}$$

Where D(*NN*) represents the nearest neighbor distance:


$$\begin{array}{c} D\left(NN\right)=\sum_{i}^{n}\frac{min\left(d_{ij}\right)}{N} \end{array}$$

D(ran) represents the theoretical average distance under a random distribution:


$$\begin{array}{c} D\left(ran\right)=0.5\sqrt{\frac{A}{N}} \end{array}$$

Where N represents the number of sample points, which corresponds to the number of settlement sites; $d_{ij}$ denotes the distance between the i-th point and the j-th point; $min\left(d_{ij}\right)$ indicates the distance from the i-th point to its nearest neighbor, and A signifies the area of the study region. Generally, NNI < 1 indicates clustering (NNI = 0 denotes perfect clustering), NNI = 1 indicates a random distribution, and NNI > 1 indicates dispersion. A Z-value test (p < 0.01) was used to assess statistical significance. NNI is used here solely to confirm the presence of clustering, not as a linear measure of aggregation intensity due to its sensitivity to sample size and density changes.

#### 3.2.2. Kernel density analysis method.

Kernel density analysis in ArcGIS 10.8 was used to examine the spatial distribution and clustering of settlements. This method employs color intensity to represent site density, with darker shades indicating higher-density areas and lighter shades indicating more dispersed patterns. Based on the distance-decay law, closer distances are assigned greater weights to statistically and visually characterize spatial density within the study area [30]. Specifically, areas with denser point distributions have a higher probability of events than regions with sparser distributions. The definition of kernel density estimation is as follows: Let x₁, x₂,..., xₙ be independent and identically distributed samples drawn from a population with an unknown distribution density function, f. To estimate the value of the density function, f(x), at a specific point x, the Rosenblatt-Parzen kernel estimation method was used:


$$\begin{array}{c} f_{n}\left(x\right)=\frac{\text{1}}{nh}\sum_{i=1}^{n}k\left(\frac{x-X_{i}}{h}\right) \end{array}$$

Where k () is referred to as the kernel function; h > 0 denotes the bandwidth; (x - X_i_) represents the distance between the evaluation point x and the data point X_i_. The output range was unified (0–0.4 sites/km²) across all phases for cross-period comparison.

#### 3.2.3. Calculation of least-cost path distances between settlements.

Euclidean distance considers only straight-line spatial separation and disregards the obstructive or facilitating effects of terrain and hydrological features on prehistoric human movement. To better reflect actual connectivity, this study adopts a cost‑surface weighted Euclidean distance approach.

To account for the influence of terrain, hydrological networks and lakes on settlement connectivity, factors such as elevation, slope, topographic relief and water systems were classified and scores assigned to reflect their relative impact on movement. The Analytic Hierarchy Process (AHP) [31] was used to determine the weight of each factor through structured expert elicitation (Table 1).Five researchers specializing in archaeology and geomorphology independently completed pairwise comparison matrices for elevation, slope, terrain undulation, and water-system hierarchy. The geometric mean of their responses was used to derive the final weights. Consistency Ratios (CR) were calculated for all matrices; all CR values were ≤ 0.08, indicating acceptable internal consistency.

**Table 1:** Weight of geographical elements and cost values.

Geographical elements	Weight	Grade	Cost value
Altitude(m)	0.4156	<50	1
		50-100	2
		100-200	3
		200-400	5
		400-700	10
		700−1,000	15
		1,000-1,500	20
		>1,500	25
Slope(°)	0.3428	0-3	1
		3-5	5
		5-10	10
		10-15	15
		15-25	20
		>25	25
topographic relief	0.1453	0-20	1
		20-50	5
		50-100	10
		>=100	15
Level of water system	0.0963	Level 1	1
		Level 2	5
		Level 3	10
		Level 4	15
		Level 5	20

Cost values for each factor were assigned based on modern analog studies and archaeological evidence: Altitude: < 50 m (flood risk) and >1500 m (harsh environment) assigned high costs; 100–200 m (gentle terraces) assigned low costs. Slope: 0–3° (suitable for agriculture) assigned lowest cost; > 25° (difficult for cultivation/movement) assigned highest cost. Terrain undulation: < 20 m (flat areas) assigned low cost; ≥ 100 m (rugged terrain) assigned high cost. Water system level: Level 1 (small tributaries) assigned lowest cost; Level 5 (main rivers) assigned higher cost.

**Fig 2:**
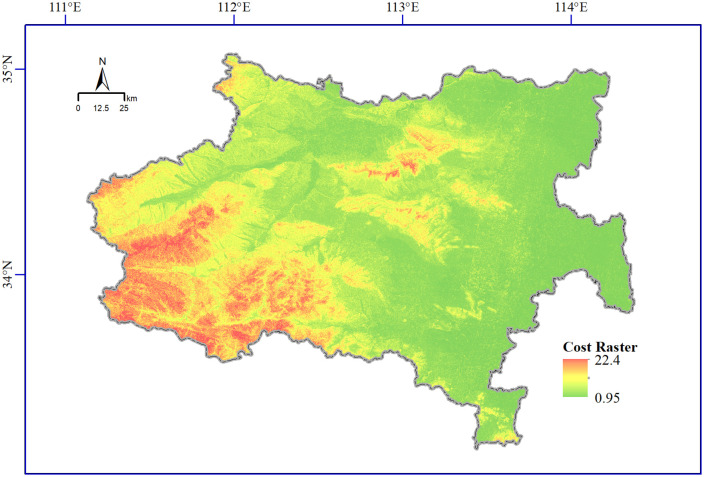
Cost raster map in the Songshan Mountain region. (Note: The cost value ranges from 0.95 to 22.4, with higher values indicating greater movement resistance).This figure was created by the authors in ArcGIS 10.8. Digital Elevation Model (DEM) data were obtained from the Geospatial Data Cloud site, Computer Network Information Center, Chinese Academy of Sciences (http://www.gscloud.cn).Administrative boundaries were derived from OpenStreetMap (https://www.openstreetmap.org) © OpenStreetMap contributors, available under the Open Database License (ODbL).

A weighted-overlay analysis in ArcGIS 10.8 Raster Calculator generated the cost surface (Fig 2). The grid cell size is 30 m × 30 m, consistent with the DEM. Sensitivity analysis with 10% perturbations to weights and cost thresholds showed <5% changes in cluster boundaries, confirming model robustness.

To realistically model the connectivity between prehistoric settlements, we used least-cost path (LCP) distances instead of Euclidean distances. A cost surface (30 m resolution) was first generated by weighted overlay of elevation, slope, topographic relief and water system hierarchy (Table 1; Fig 2). To balance accuracy and computational efficiency, the cost surface was resampled to 500 m resolution using bilinear interpolation. All subsequent calculations were performed on this resampled cost raster.

Pairwise LCP distances between all settlements were then computed using Dijkstra’s algorithm implemented in the gdistance package (v1.6.5) in R 4.6.0. The algorithm operates on a graph where each grid cell of the resampled 500 m cost raster is a node, edges connect neighboring cells (8-direction neighborhood), and edge weights are defined as the average cost value of the two adjacent cells. The shortest path between any two settlement sites is the path that minimizes the sum of edge weights along the route, i.e., the cumulative movement cost traversing the cost surface. The resulting distance matrix is symmetric and contains the actual least-cost path distance between every pair of settlements. This matrix was used as the input for clustering. The complete R code for LCP distance calculation and subsequent clustering is provided in the Supporting Information (S2 File).The least-cost path distances are expressed in cost units, where one cost unit represents the weighted impedance of a single 500 m × 500 m grid cell. Higher values indicate greater cumulative movement difficulty.

#### 3.2.4. The method of settlement cluster division.

We applied k-medoids clustering (specifically the Partitioning around Medoids, PAM) to the LCP distance matrix. K-medoids is more robust to outliers than k-means and selects actual sites as cluster centers (Medoids), which is particularly suitable for archaeological distance matrices [32].

The optimal number of clusters k for each period was determined by maximizing the average silhouette width. For k ranging from 2 to min (10, n − 1) where n is the number of sites, we performed k-medoids clustering and calculated the average silhouette width of the resulting partition. The k with the highest average silhouette width was selected as the optimal cluster number. This procedure yielded optimal cluster counts of k = 9 (average silhouette width = 0.48) for the Peiligang period, k = 5 (0.52) for the Yangshao period, k = 4 (0.50) for the Longshan period, and k = 2 (0.57) for the Xia-Shang period. Final clustering for each period was performed with the selected k using the pam function in R (random seed = 42). Stability was assessed by 20 independent runs with random seeds 1–20; all Jaccard similarity coefficients between each run and the benchmark run (seed = 42) were ≥ 0.92, confirming consistent cluster configurations. The complete R script and output files are provided in the Supporting Information.

## 4. Results

### 4.1. Temporal evolution of settlement agglomeration

Using the Average Nearest Neighbor Distance tool in ArcGIS 10.8 spatial distribution pattern suite, we measured the distance from each element to its nearest neighbor and calculated the mean observed distance. The tool then compared this mean to the expected distance under a random distribution and computed a Z-score representing its deviation. Negative Z-scores indicate clustering, whereas positive values indicate dispersion. The results are presented in Table 2.

**Table 2:** Analysis of the average nearest neighbor distance of settlements from the Peiligang to Xia-Shang period in the Songshan Mountain region.

Periods	Number of samples	Average nearest distance (m)	Expected average nearest distance (m)	NNI statistics	Z-test statistic	P value
Peiligang	118	5,566.96	8,577.63	0.60	−8.02	0.00
Yangshao	563	2,648.93	3,975.95	0.67	−15.32	0.00
Longshan	660	2,242.07	3,672.17	0.61	−19.14	0.00
Xia-Shang	634	2,086.62	3,746.71	0.56	−21.34	0.00

As shown in Table 2, the NNI values for settlements in the Songshan Mountain area during all four periods are less than 1 and have passed the significance test at p < 0.01, confirming a persistent clustered spatial distribution. All periods exhibit statistically significant clustering, with NNI values ranging from 0.56 (Xia-Shang) to 0.67 (Yangshao).

### 4.2. Characteristics of spatial agglomeration in settlements

**Fig 3:**
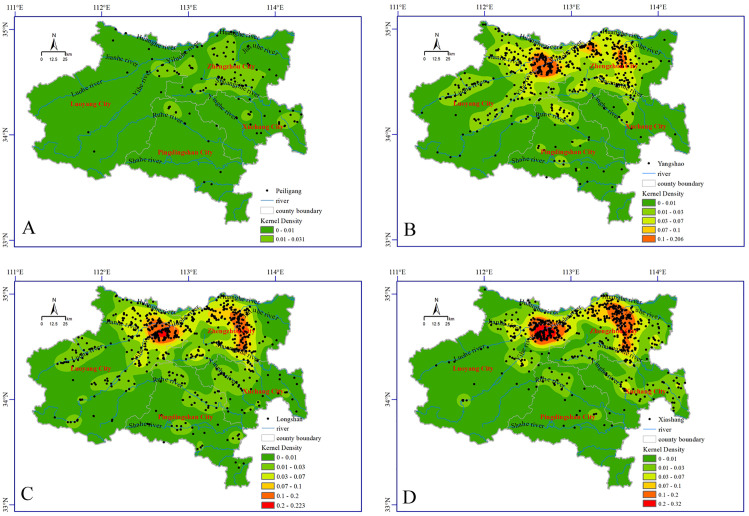
Settlement Kernel Density Distribution Maps from the Peiligang to the Xia-Shang period in the Songshan Mountain region: (A) Peiligang; (B) Yangshao; (C) Longshan; (D) Xia-Shang period. This figure was generated by the authors in ArcGIS 10.8 using the authors’ compiled settlement site dataset (as detailed in Section 3.1). Administrative boundaries and hydrological network data were obtained from OpenStreetMap (https://www.openstreetmap.org), © OpenStreetMap contributors, available under the Open Database License (ODbL).

The Kernel Density Analysis tool in ArcGIS 10.8 was employed to quantify the spatial clustering characteristics of settlements for each period, using a uniform output range of 0–0.4 sites/km² (Fig 3). The kernel density analyses presented in Fig 3 were conducted using the full settlement dataset (118, 563, 660, and 634 sites for each respective period).

The bandwidth for the kernel density analysis was set at 5 km for the following reasons: (1) the average distance between settlements in the study area is approximately 4.8 km, calculated from the coordinates of 1,975 archaeological sites. A bandwidth slightly greater than this average helps avoid excessive fragmentation of density clusters. (2) Sensitivity analyses indicate that varying the bandwidth between 4 and 6 km results in minimal changes to the spatial distribution of the core high-density regions, as evidenced by a Jaccard coefficient of ≥0.95. Only minor fluctuations in density values occur at peripheral areas, which do not influence the primary conclusions of the analysis. By integrating density gradient patterns with geographic coordinates, the spatio-temporal evolution pattern of settlement agglomeration was revealed through quantitative analysis.

The distribution of settlement sites during the Peiligang period was relatively dispersed, with a single, small high-density kernel centered around Zhengzhou at 0.031 sites per square kilometer (Fig 3A). Additional low-density clusters were scattered near Luoyang and Xuchang, forming a pattern of multiple small-scale aggregations distributed along the periphery of the Songshan Mountain region. During the Yangshao period (Fig 3B), compared to the Peiligang period, the spatial extent of settlements expanded significantly, and overall density increased markedly. The kernel density high-value zone shifted from the eastern part of Songshan Mountain to the northern Luoyang area, where the peak density reached 0.206 sites/km²—an increase of 500.64% compared to the Peiligang period. In the Longshan period (Fig 3C), settlement density further increased, and the high density zone expanded and became more concentrated. Luoyang remained the area with the highest settlement concentration, with a peak value of 0.223 sites/km², representing an 8.25% increase over the Yangshao period. Additionally, settlement densities in regions such as eastern Zhengzhou (Jialu River–Shuangji River Basin) and northern Xuchang (Ying River Basin) also rose to varying degrees. During the Xia-Shang period (Fig 3D), settlement density continued to rise, reaching a maximum of 0.316 sites/km²—the highest value across the entire sequence—an increase of 41.7% compared to the Longshan period. A spatial pattern consistent with a dual-core structure can be observed, with settlements concentrated around the two major centers at Yanshi and Zhengzhou.

The number of settlement sites in the Songshan Mountain region exhibited a consistent upward trend from the Peiligang to the Xia-Shang period. While the spatial extent of settlements continued to expand, settlement density also increased progressively. Throughout this period, site distribution remained centered on Songshan Mountain and its adjacent river valleys. The core aggregation zone shifted across phases: from the Zhengzhou area during the Peiligang period, to the Luoyang area in the Yangshao and Longshan periods, and finally evolving into a “dual-core” spatial pattern encompassing both Zhengzhou and Luoyang by the Xia-Shang period.

### 4.3. Division of settlement clusters

**Fig 4:**
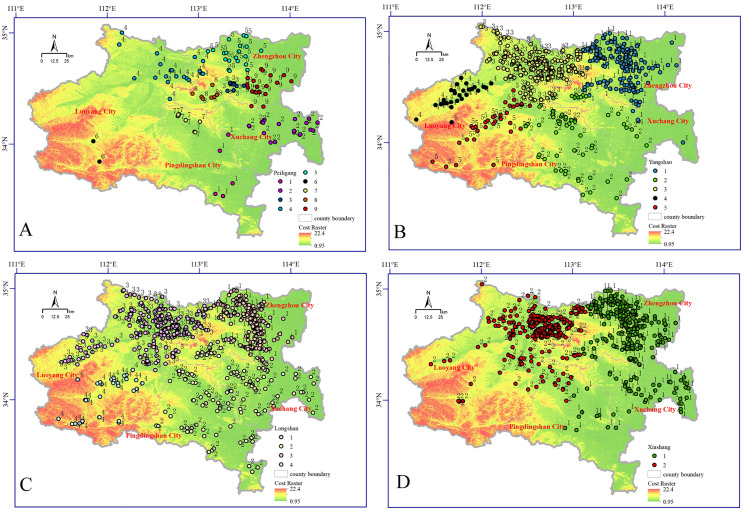
Division of settlement clusters from the Peiligang to the Xia-Shang period in the Songshan Mountain region: (A) Peiligang; (B) Yangshao; (C) Longshan; (D) Xia-Shang. This figure was created by the authors in ArcGIS 10.8. Digital Elevation Model (DEM) data were obtained from the Geospatial Data Cloud site, Computer Network Information Center, Chinese Academy of Sciences ([href:http://broken-link/]http://www.gscloud.cn). Administrative boundaries were derived from OpenStreetMap (https://www.openstreetmap.org), ©OpenStreetMap contributors, available under the Open Database License (ODbL).

The optimal number of k-medoids clusters decreases markedly through time: from 9 in the Peiligang period to 5 in Yangshao, 4 in Longshan, and 2 in the Xia-Shang period (Table 3; Fig 4). These clustering analyses were performed on the effective sample sizes after excluding sites that fell outside the continuous cost surface or lacked valid coordinates for LCP calculation (Peiligang: 117 sites; Yangshao: 561; Longshan: 659; Xia-Shang: 632; see Section 3.1).This trend, which is opposite to the increasing number of settlements, suggests a progressive integration of cost connectivity networks. Table 3 summarizes the within cluster least-cost path distances (in cost units) for each period and cluster.

**Table 3:** Settlement count, proportion, and within-cluster least-cost path distances by period and cluster.

Period	Cluster ID	Number of sites	% of total	Min-distance	Mean-distance	Max-distance
Peiligang	1	5	4.3	18,272.51	68,149.28	119,037.10
	2	19	16.2	712.85	47,742.66	118,576.30
	3	12	10.3	1,480.48	22,084.01	58,957.57
	4	22	18.8	1,837.20	109,627.50	320,443.50
	5	20	17.1	4,892.62	52,014.67	118,523.30
	6	2	1.7	180,179.80	180,179.8	180,179.80
	7	9	7.7	0	37,635.26	75,936.22
	8	7	6	8,633.98	50,624.26	104,249.30
	9	21	17.9	0	47,964.60	118,878.50
Yangshao	1	194	34.6	0	89,144.67	330,979.30
	2	63	11.2	0	137,895.60	342,538.90
	3	239	42.6	0	92,651.19	468,622.50
	4	29	5.2	4,662.62	103,411.60	389,459.00
	5	36	6.4	0	225,505.00	839,149.20
Longshan	1	211	32	0	72,508.69	217,888.70
	2	118	17.9	1,457.09	135,278.10	364,624.60
	3	297	45.1	0	115,417.30	433,147.10
	4	33	5	0	306,287.20	819,718.40
Xia-Shang	1	325	51.4	0	103,968.20	401,154.90
	2	307	48.6	0	112,157.90	725,002.60

During Peiligang period, the nine clusters are highly uneven in size and internal connectivity (Fig 4A). Cluster 4 (22 sites, 18.8%) occupies the Yi-Luo River Basin, with a mean within cluster distance of 109,627.5 cost units (min = 1,837.2, max = 320,443.5), indicating a wide internal cost range. Cluster 9 (21 sites, 17.9%), located in southern Zhengzhou, shows a mean distance of 47,964.6 cost units (min = 0, max = 118,878.5). Cluster 5 (20 sites, 17.1%) lies along the northeastern piedmont of Songshan Mountain; its mean distance is 52,014.67 cost units (min = 4,892.62, max = 118,523.3). Cluster 2 (19 sites, 16.2%) in the Xuchang Shuangji River Basin has a mean distance of 47,742.66 cost units (min = 712.85, max = 118,576.3). The three smallest clusters—Cluster 1 (Sha River Basin, 5 sites), Cluster 3 (southeastern Songshan Mountain, 12 sites) and Cluster 7 (Ru River Basin, 9 sites) have mean distances of 68,149.28, 22,084.01 and 37,635.26 cost units respectively. Cluster 8 (southern Songshan Mountain, 7 sites) has a mean distance of 50,624.26 cost units (min = 8,633.98, max = 104,249.3). The smallest group, Cluster 6 (upper Yi River, 2 sites), has a single within cluster distance of 180,179.8 cost units, reflecting its isolated location. The high variation in internal distances suggests that the nine Peiligang groups had very different levels of internal integration.

During Yangshao period, the five clusters show a more balanced distribution (Fig 4B). Cluster 3 (239 sites, 42.6%), centered in the Yi-Luo River Basin, has a mean within cluster distance of 92,651.19 cost units (min = 0, max = 468,622.5). Cluster 1 (194 sites, 34.6%), occupying the Jialu River area around Zhengzhou, has a mean distance of 89,144.67 cost units (min = 0, max = 330,979.3). Cluster 2 (63 sites, 11.2%) in the Pingdingshan and Xuchang region has a mean distance of 137,895.6 cost units, the highest among the five clusters, indicating that sites in this group were more dispersed. Cluster 5 (36 sites, 6.4%) in the upper Yi River valley has the largest mean distance (225,505 cost units) and the largest maximum (839,149.2), suggesting a highly elongated or fragmented distribution. Cluster 4 (29 sites, 5.2%) in the Luo River Basin shows a mean distance of 103,411.6 cost units. The reduction from 9 to 5 clusters and the generally lower internal distances (except for the peripheral groups) suggest a coalescence of previously separate groups into larger, better connected networks.

During Longshan period, further reduction to four clusters (Fig 4C). Cluster 3 (297 sites, 45.1%) remains dominant in the Yi-Luo River Basin, with a mean within cluster distance of 115,417.3 cost units (min = 0, max = 433,147.1). Cluster 1 (211 sites, 32.0%) in the Zhengzhou Jialu River area has a mean distance of 72,508.69 cost units (min = 0, max = 217,888.7), the lowest among all Longshan clusters. Cluster 2 (118 sites, 17.9%) in the Pingdingshan and Xuchang region has a mean distance of 135,278.1 cost units (min = 1,457.09, max = 364,624.6). Cluster 4 (33 sites, 5.0%) in the upper Yi River valley shows a very high mean distance of 306,287.2 cost units (min = 0, max = 819,718.4), indicating that this small group consists of widely dispersed sites. The persistence of the four groups under increasingly cooler and drier conditions, combined with the relatively low internal distances in the core clusters (Clusters 1 and 3), suggests that communities aggregated into more resilient cost connectivity networks.

During Xia-Shang period, a striking bipolar structure with two clusters of nearly equal size (Cluster 1: 325 sites, 51.4%; Cluster 2: 307 sites, 48.6%; Fig 4D). Cluster 1 (western foothills of Songshan Mountain, Luoyang area) has a mean within cluster distance of 103,968.2 cost units (min = 0, max = 401,154.9). Cluster 2 (eastern foothills of Songshan Mountain, Zhengzhou area) has a slightly higher mean distance of 112,157.90 cost units (min = 0, max = 725,002.6). The almost equal split and the comparable internal distances suggest a cluster configuration consistent with what might be expected if an early state society had contributed to regional polarization, creating two major cost connectivity domains of similar internal integration and superseding the earlier multi-center pattern.

It is important to emphasize that these clusters are descriptive spatial constructs generated independently for each period using the k-medoids algorithm. They are not assumed to represent persistent socio-political entities with continuity through time. Cross-period comparisons refer to the spatial persistence of high-density areas and the similarity of cluster distribution patterns, not the evolution of specific social groups or political territories.

## 5. Discussion

**Fig 5:**
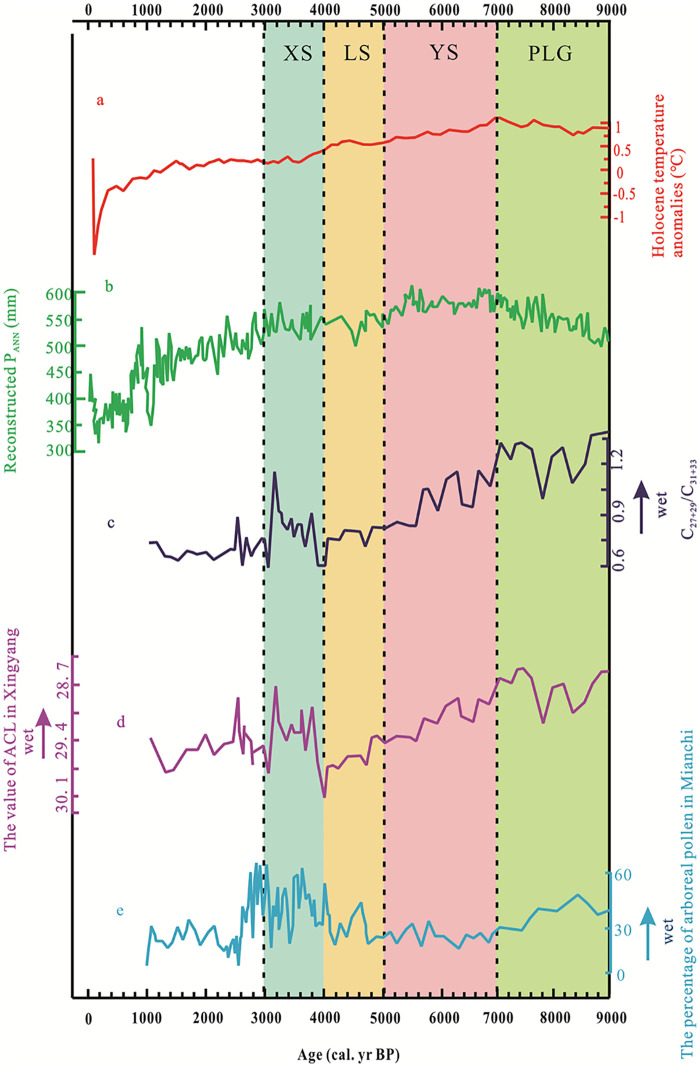
The changes in precipitation, temperature and pollen indicators in the study area since 9,000 years ago. (a) Simulated mean temperatures based on mollusks from East Asia [24]. (b) Pollen-based annual precipitation (PANN) reconstructed from Gonghai Lake [25]. (c) C_27+29_/C_31+33_ ratio in the Xingyang Basin [26] (d) ACL in Xingyang basin [26]. (e) The percentage of arboreal pollen from the Mianchi Basin [27]. This composite figure was created by the authors based on data from the cited sources.

The findings indicate that from the Peiligang Period to the Xia-Shang period, settlements in the Songshan Mountain region consistently exhibited a pronounced clustered distribution pattern. This pattern persisted throughout the entire study period, constituting a core characteristic of the spatial organization of prehistoric human activity in the region. Furthermore, this enduring clustered pattern shows a strong correspondence with the phased climatic evolution of the area over the past approximately 9,000 years (Fig 5). Building upon the descriptive spatial framework established in Section 4.3, we now examine the relationship between these settlement patterns and environmental change.

### 5.1. Explicit interpretation of paleoclimate context and resolution of discrepancies

The analysis of paleoclimate data provides the environmental context for understanding this aggregation behavior. The temperature trends are derived from regional and global temperature reconstructions based on multiple proxy records [24] (curve a),which show a gradual warming trend from the Peiligang to the Yangshao period, reaching a peak during the Holocene Thermal Maximum, followed by a gradual cooling trend from the Longshan to the Xia-Shang period. This temperature evolution provides the fundamental climatic background for the transformation of prehistoric livelihood economy and settlement distribution.

Crucially, hydrological conditions are interpreted from multiple complementary proxy sources with a clear interpretative rationale, and the apparent discrepancies between different climate curves are resolved by focusing on absolute humidity levels and overall climatic stage characteristics rather than single directional trends:Curve b (pollen-based annual precipitation from Gonghai Lake [25]) shows an increase in regional average precipitation from the Peiligang to the Yangshao period, reflecting the overall wetter climatic stage of the Holocene Thermal Maximum; Curves c and d (n-alkane indices from the Xingyang Basin [26]) show fluctuating humidity during the Yangshao period, with short-term dry phases but the absolute humidity level consistently above the two prominent dry minima in the Peiligang period; Pollen data from the Mianchi Basin [27] (Curve e) further confirms regional vegetation succession: the increase in arboreal and hydrophilic plant pollen during the Yangshao period aligns with enhanced precipitation, while the decline in the Longshan-Xia-Shang period corresponds to cooler and drier conditions.

In summary, the Yangshao period is an overall warm and wet climatic stage compared to the Peiligang period, and the discrepancies between the curves reflect the difference between regional average precipitation (curve b) and local humidity fluctuations (curves c/d), rather than contradictory climatic trends. This nuanced interpretation resolves the apparent conflict and provides a consistent climatic context for analyzing settlement and livelihood economy evolution.

The synthesized trends presented in Fig 5 include pollen-based vegetation/humidity indicators and do not represent precise, point-specific measurements but illustrate the overall directional shifts in temperature, moisture availability and vegetation cover that formed the background for human settlement decisions and livelihood economy transformation.

### 5.2. Coupling of settlement cluster evolution, climate change and prehistoric livelihood economy

During Peiligang period (9000–7500 BP), the East Asian summer monsoon gradually intensified, with increasing temperature and precipitation [24–27]. The warm and humid climate coincided with favourable conditions for the emergence of agriculture, though hunting, gathering and fishing remained dominant [33]. Settlement patterns were likely constrained by topography, forming small aggregations along foothills and river valleys. The nine least-cost path clusters were highly uneven, suggesting that natural resource heterogeneity strongly influenced early settlement distribution.

During Yangshao period (7500–5000 BP), the region experienced the Holocene Thermal Maximum, with temperatures 1–2 °C higher and precipitation 30% greater than today [24–27]. Agricultural advances, especially millet cultivation, enabled population expansion into plains. The favourable climate was associated with a 3.7-fold increase in settlement number. Habitable space expanded from foothills to plains, leading to a more balanced cluster distribution compared to Peiligang. Notably, the Ying River Basin was the only area with a slight decrease in settlements, possibly related to local river siltation under high precipitation [26].

During the Longshan period (5000–4000 BP), the climate transitioned toward cooler conditions compared to the Yangshao period [24]. Multiple paleoclimate proxies from the study area suggest increasing moisture variability and gradual vegetation degradation [26,27], although regional precipitation reconstructions indicate that humidity may have remained relatively high in some areas [25]. The predictability and spatial distribution of water resources likely changed, posing challenges for agricultural societies. Despite these environmental constraints, the total number of settlements increased to 660, and the number of k-medoids clusters reduced from 9 (Peiligang) to 5 (Yangshao) to 4 (Longshan).This reduction suggests that communities increasingly aggregated into larger cost-connectivity networks, possibly in response to resource competition and inter-group cooperation. The Yi-Luo and Jialu-Shuangji plains remained the core areas, accounting for 74% of all Longshan sites.

During the Xia-Shang period (4000–3000 BP), the Holocene Thermal Maximum waned and climatic conditions continued to cool [24]. Multiple proxies indicate a trend toward increased aridity and reduced vegetation cover across the region [26,27], although the magnitude and timing of precipitation changes varied spatially [25]. However, settlement aggregation intensity reached its peak, forming a dual-core polarized pattern (k = 2). This apparent contradiction indicates a decoupling from direct climatic control: socio-political factors, particularly the emergence of early states centered at Yanshi and Zhengzhou became the dominant force shaping settlement distribution. A four-tier hierarchical settlement system (capitals, large/mid-size urban sites, small urban sites, ordinary settlements) had been established [34]. The spatial configuration is consistent with a scenario in which political factors became increasingly influential, potentially superseding climatic suitability as the dominant organizing principle. Peripheral areas (e.g., upper Luo and Yi Rivers) contracted sharply as population and resources were drawn toward the two cores. This transition can be interpreted as a qualitative shift in human-environment relationships: from a pattern more strongly associated with environmental factors to one in which socio-political factors appear to have become increasingly influential, even under deteriorating climatic conditions.

## 6. Conclusions

Settlements, as core carriers of early human activities, exhibit agglomeration characteristics that serve as the basis for identifying and delineating settlement clusters. Therefore, verifying whether settlements display an aggregated distribution pattern constitutes a fundamental step in cluster division research. This study establishes a systematic technical framework for delimiting prehistoric settlement clusters, following a “three-step progressive” logic: first, assessing the overall spatial aggregation of regional settlements through nearest neighbor distance analysis; second, revealing the spatial differentiation patterns of agglomeration using kernel density analysis; and finally, integrating the least-cost path model with the K-medoids algorithm to precisely define cluster boundaries and analyze their evolutionary dynamics. Applying this framework to settlement data from the Neolithic through the Xia-Shang period in the Songshan Mountain region has deepened understanding of the spatiotemporal evolution of human-environment interactions in the core area of early Chinese civilization and provides critical evidence for investigating the origins of civilization and the development of social complexity. The main conclusions are as follows:

(1) The temporal characteristics of settlement agglomeration: Across the Peiligang, Yangshao, Longshan, and Xia-Shang period, settlements in the Songshan Mountain region consistently exhibited a significant aggregated distribution.
(2) Spatial evolution of the core agglomeration area: The high-density core zone of settlements exhibits a clear trend of westward shift. During the Peiligang period, the core was concentrated in the eastern part of Songshan Mountain (centered on Zhengzhou), developing around the favorable water resources and gentle terrain of the Jialu River–Shuangji River Basin. From the Yangshao to the Longshan periods, the core shifted northward to the northern flank of Songshan Mountain, where the Yi-Luo River Basin gradually emerged as the new center, while development in the eastern core continued. By the Xia-Shang period, a persistent spatial pattern that can be interpreted as a potential dual-core structure had formed centered on Yanshi and Zhengzhou. This spatial evolution reflects the progressive expansion of human settlement space and the ongoing optimization of site selection strategies driven by environmental adaptation.
(3) From the Peiligang to the Xia-Shang period, the optimal number of settlement clusters exhibited a clear downward trend, declining from 9 to 5 (Yangshao), 4 (Longshan), and finally to 2 (Xia-Shang). Despite more than five-fold increase in total settlement number, the clustering structure simplifies, suggesting a progressive coalescence of interaction networks and the emergence of a spatial pattern that is consistent with a polarized socio-political landscape. In the Peiligang period, the clusters were highly uneven, with a single large cluster and many small ones; by the Xia-Shang period, the bipolar pattern reflects the dual-core state organization.
(4) Overall, from the Peiligang to the Longshan period, environmental factors (topography, water resources, and climate) are correlated with observed settlement patterns, suggesting that settlement patterns were more strongly associated with environmental factors. During the Xia-Shang period, however, despite cooler and drier climatic conditions, socio-political factors appear to have become increasingly important, possibly overriding direct environmental constraints. This transition from a pattern more strongly associated with environmental factors to one in which socio-political factors appear to have become increasingly influential can be viewed as a key threshold in the evolution of human–environment interactions in the core area of early Chinese civilization.

## Supporting information

S1 File Analytical parameters and detailed methodological procedures. (AHP weights, cost thresholds, kernel density bandwidth, and detailed methodological procedures for least‑cost path calculation, optimal k selection, k-medoids clustering, and stability tests.).
(PDF)

S2 File R analysis script. (Executable R script (version 4.6.0) implementing all steps: reading data, computing least-cost path distances, determining optimal k, performing k-medoids clustering, and exporting results. The script uses the raster, gdistance, sf, cluster, and clue packages.).
(ZIP)

S3 File Cluster membership tables. (CSV format, packaged as ZIP. Contains cluster assignments for all four cultural periods: Peiligang, Yangshao, Longshan, and Xia-Shang.).
(ZIP)

S4 File Cost surface rasters. (Raster datasets for least-cost path modeling, packaged as ZIP. Includes the original cost surface at 30 m resolution and the resampled cost surface at 500 m resolution in GeoTIFF format.).
(ZIP)
