# Supplementary material for: Spatiotemporal evolution and clustering patterns of settlements from the Neolithic to the Bronze Age (9000–3000 BP) in the Songshan Mountain region, China
Source: PLoS One. 2026 Jul 22;21(7):e0351644. doi: 10.1371/journal.pone.0351644 (PMC13390836; doi:10.1371/journal.pone.0351644)
Supplement: S1 File — (AHP weights, cost thresholds, kernel density bandwidth, and detailed methodological procedures for least‑cost path calculation, optimal k selection, k-medoids clustering, and stability tests.). (PDF) [file pone.0351644.s001.pdf]

This document provides the complete analytical parameters, computational details, and supplementary results that support the main manuscript. All analyses were performed using R version 4.6.0 (packages: `raster`, `gdistance`, `sf`, `cluster`, `clue`) and ArcGIS 10.8.

1. Data Preparation

1.1 DEM and Derived Geospatial Data

- Source: Geospatial Data Cloud (http://www.gscloud.cn).
- Product: ASTER GDEM v3, 30 m resolution.
- Processing:
    - Slope was derived using the Slope tool in ArcGIS.
    - Topographic relief was calculated using Focal Statistics with a 1 km circular neighborhood.
    - The hydrological network was extracted using the ArcGIS Hydrology toolbox (Fill, Flow Direction, Flow Accumulation, Stream Order).

1.2 Settlement Site Data

- Data sources: China Cultural Relics Map Series – Henan Volume, Henan Province Cultural Relics Annals, Third National Cultural Relics Survey.
- Georeferencing: Coordinates were collected via the Baidu Map API and projected to WGS_1984_UTM_Zone_49N for spatial analysis.
- Attribute database: Site_ID, cultural period (Peiligang/Yangshao/Longshan/Xia-Shang), site area (ha), cultural layer thickness (cm), and representative artifacts.

1.3 Paleoclimate Proxy Data

- Temperature: Regional composite curve based on East Asian mollusk fossils (Marcott et al., 2013).
- Precipitation: Pollen-based annual precipitation from Gonghai Lake (Chen et al., 2015).
- Local humidity and vegetation: n-alkane indices (C_{27+29}/C_{31+33} and ACL) from the Xingyang Basin (Liu et al., 2024).

2. Settlement Aggregation Analysis (Nearest Neighbor Analysis)
- Tool: Average Nearest Neighbor (ArcGIS Spatial Statistics Toolbox).
- Distance method: Euclidean distance.

- Interpretation: The Nearest Neighbor Index (NNI) was used only to confirm significant clustering (NNI < 1, $p < 0.01$), not as a quantitative measure of clustering intensity.

3. Kernel Density Estimation (KDE)

- Tool: Kernel Density (ArcGIS Spatial Analyst Toolbox).
- Unified parameters across all periods:
  - Output cell size: 100 m
  - Search radius (bandwidth): 5000 m
  - Area units: square kilometers
  - Normalization: All rasters scaled to 0–0.4 sites/km^2 for consistent cross-period comparison.

4. Cost Surface Construction

The cost surface was generated using weighted overlay of four terrain and hydrological factors. Weights were derived using the Analytic Hierarchy Process (AHP) based on expert pairwise comparisons.

4.1 AHP Weights and Consistency

- Altitude: 0.4156
- Slope: 0.3428
- Topographic relief: 0.1453
- Water system level: 0.0963
- Consistency ratio (CR): 0.03 (< 0.1, acceptable consistency).

4.2 Cost Surface Generation

- Weighted Sum tool in ArcGIS 10.8.
- Original 30 m raster resampled to 500 m using bilinear interpolation to balance accuracy and computational efficiency.
- Sensitivity analysis: 10% perturbations in weights and thresholds altered cluster boundaries by < 5%, confirming robustness.

5. Least-Cost Path (LCP) Distance Matrix Calculation

For each period, pairwise LCP distances were computed in R:

```r
library(raster)
library(gdistance)
library(sf)
cost_raster <- raster("costraster_500m.tif")
tr <- transition(cost_raster, function(x) 1/mean(x), directions = 8)
```

```
tr <- geoCorrection(tr)
sites <- st_read("settlement_shapefile.shp")
coords <- st_coordinates(sites)
cost_dist <- costDistance(tr, coords)
```

The resulting symmetric distance matrix was used for k-medoids clustering.

6. Optimal Cluster Number (k) Determination

For each period, we performed k-medoids clustering (PAM algorithm) for k = 2 up to min(10, n-1), where n is the number of sites. The **average silhouette width** (calculated with cluster::silhouette) was used as the quality criterion. The k with the highest average silhouette width was chosen as the optimal cluster number. Results:

Period	n (valid sites)	Optimal k	Average silhouette width
Peiligang	117	9	0.48
Yangshao	561	5	0.52
Longshan	659	4	0.50
Xia-Shang	632	2	0.57

Note: A few sites falling on NA cells of the cost raster were excluded (1 for Peiligang, 2 for Yangshao, 1 for Longshan, 2 for Xia-Shang).

7. Final Clustering and Stability Test

Clustering was performed using the optimal k and random seed = 42 for reproducibility. Stability was evaluated using 20 repeated runs with random seeds 1–20. Jaccard similarity was calculated using `clue::cl_agreement`.

Period	Minimum Jaccard	Mean Jaccard	Standard deviation
Peiligang	0.94	0.97	0.02
Yangshao	0.93	0.96	0.01
Longshan	0.92	0.96	0.02
Xia-Shang	0.95	0.98	0.01

8. Cluster Size Distribution

Period (optimal k)	Cluster ID	Number of sites	% of total
Peiligang (k=9)	1	5	4.3
	2	19	16.2
	3	12	10.3
	4	22	18.8
	5	20	17.1
	6	2	1.7
	7	9	7.7
	8	7	6
	9	21	17.9
Yangshao (k=5)	1	194	34.6
	2	63	11.2
	3	239	42.6
	4	29	5.2
	5	36	6.4
Longshan (k=4)	1	211	32
	2	118	17.9
	3	297	45.1
	4	33	5
Xia-Shang (k=2)	1	325	51.4
	2	307	48.6

9. Cluster Membership CSV Files (separately attached)

- Peiligang_optimal9clusters.csv
- Yangshao_optimal5clusters.csv
- Longshan_optimal4clusters.csv
- XiaShang_optimal2clusters.csv

Each file includes Site_ID and Cluster.

10. Software and Parameter Summary

**Step**	**Software / package**	**Version**	**Key parameters**
Cost surface generation	ArcGIS	10.8	AHP weights (Table 1), resampling to 500 m (bilinear)

Step	Software / package	Version	Key parameters
Least-cost path distance	R gdistance	1.6.5	transitionFunction = 1/mean(x), directions = 8, geoCorrection
K-medoids (PAM)	R cluster	2.1.4	diss = TRUE, random seed = 42
Silhouette width	R cluster	2.1.4	default
Stability test	R clue	0.3-68	cl_agreement(method = "Jaccard")

11. Sites Excluded Due to Missing Cost Values

Period	Original sites	Sites with valid cost value	Excluded
Peiligang	118	117	1
Yangshao	563	561	2
Longshan	660	659	1
Xia-Shang	634	632	2
