# Supplementary figures and images for: Spatiotemporal evolution and clustering patterns of settlements from the Neolithic to the Bronze Age (9000–3000 BP) in the Songshan Mountain region, China
Source: PLoS One. 2026 Jul 22;21(7):e0351644. doi: 10.1371/journal.pone.0351644 (PMC13390836; doi:10.1371/journal.pone.0351644)

Optimal k – Longshan

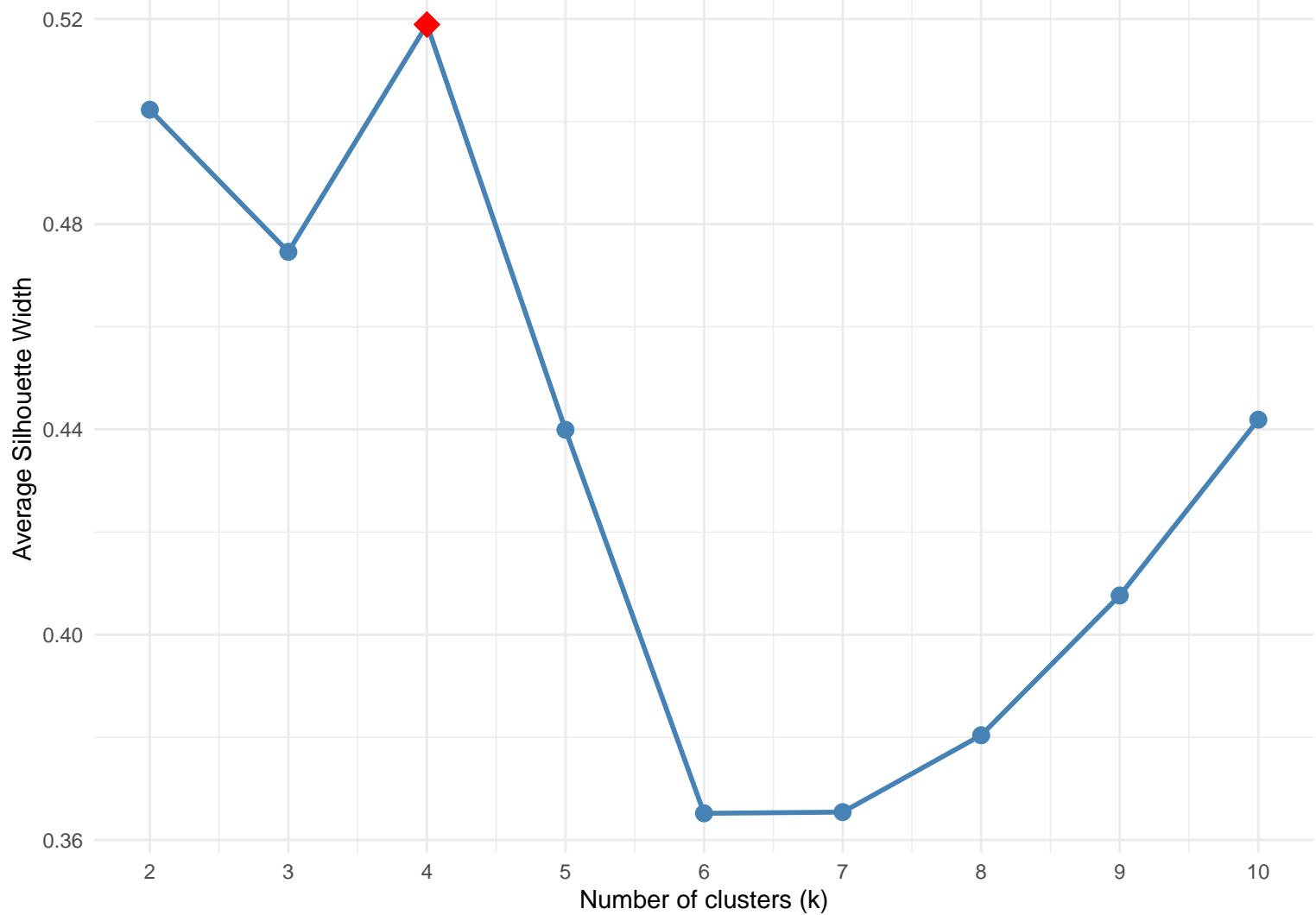

Supplement: S2 File — (Executable R script (version 4.6.0) implementing all steps: reading data, computing least-cost path distances, determining optimal k, performing k-medoids clustering, and exporting results. The script uses the raster, gdistance, sf, cluster, and clue packages.). (ZIP) [file pone.0351644.s002.zip › S2_Code/silhouette_plot_Longshan.pdf]

Optimal k – Peiligang

Average Silhouette Width

2

3

4

5

6

7

8

9

10

Number of clusters (k)

0.45

0.40

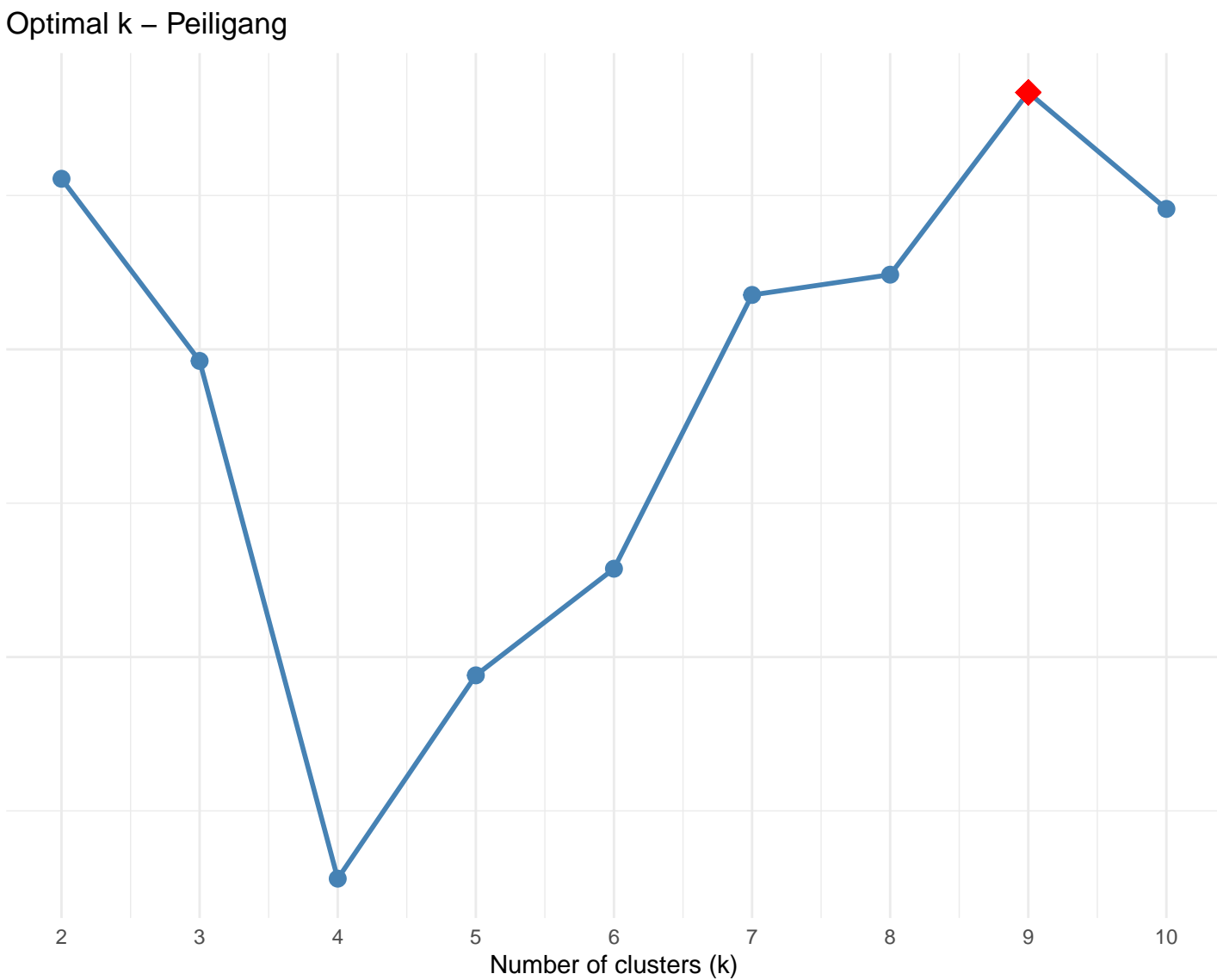

Supplement: S2 File — (Executable R script (version 4.6.0) implementing all steps: reading data, computing least-cost path distances, determining optimal k, performing k-medoids clustering, and exporting results. The script uses the raster, gdistance, sf, cluster, and clue packages.). (ZIP) [file pone.0351644.s002.zip › S2_Code/silhouette_plot_Peiligang.pdf]

Optimal k – XiaShang

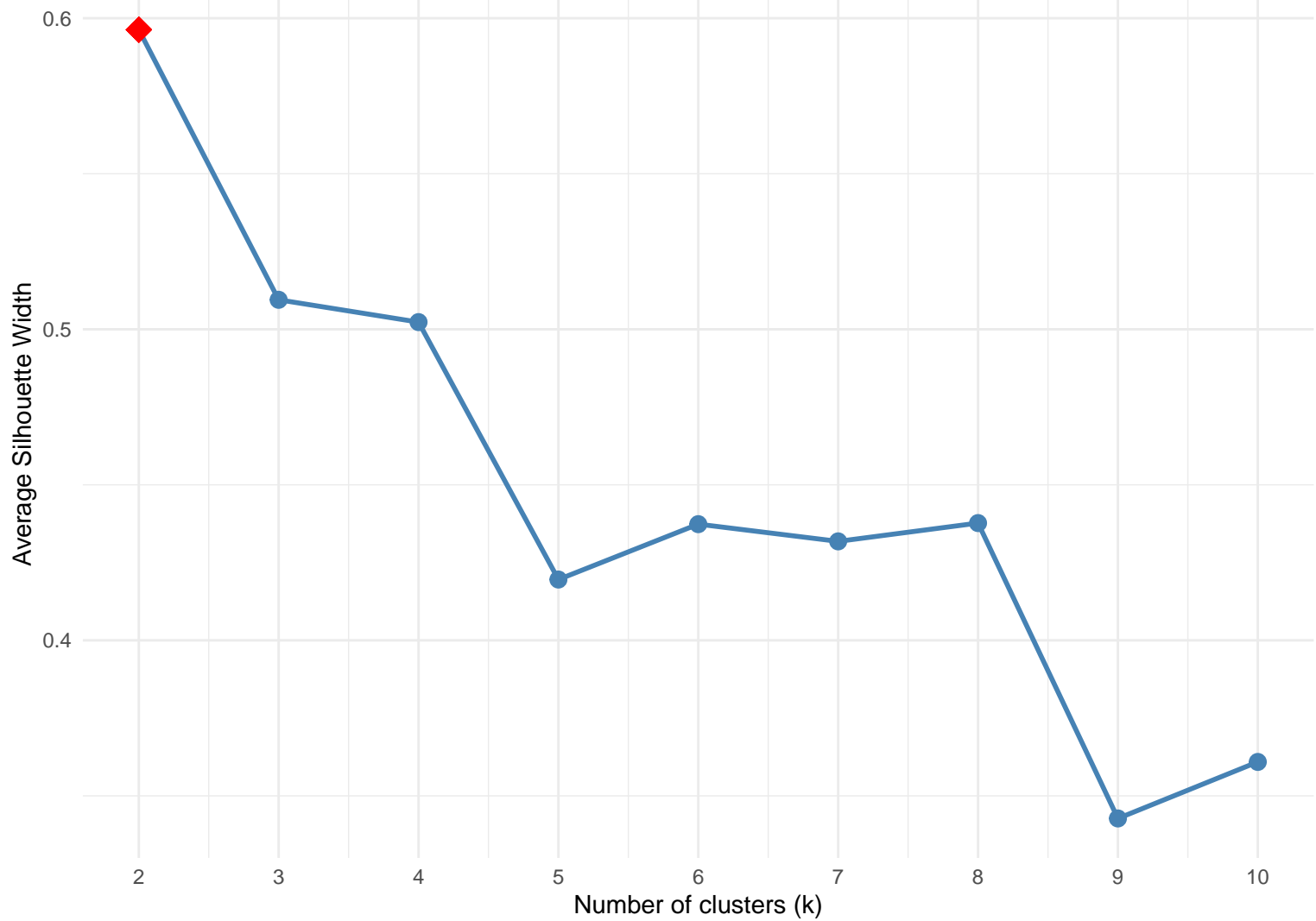

Supplement: S2 File — (Executable R script (version 4.6.0) implementing all steps: reading data, computing least-cost path distances, determining optimal k, performing k-medoids clustering, and exporting results. The script uses the raster, gdistance, sf, cluster, and clue packages.). (ZIP) [file pone.0351644.s002.zip › S2_Code/silhouette_plot_XiaShang.pdf]

Optimal k – Yangshao

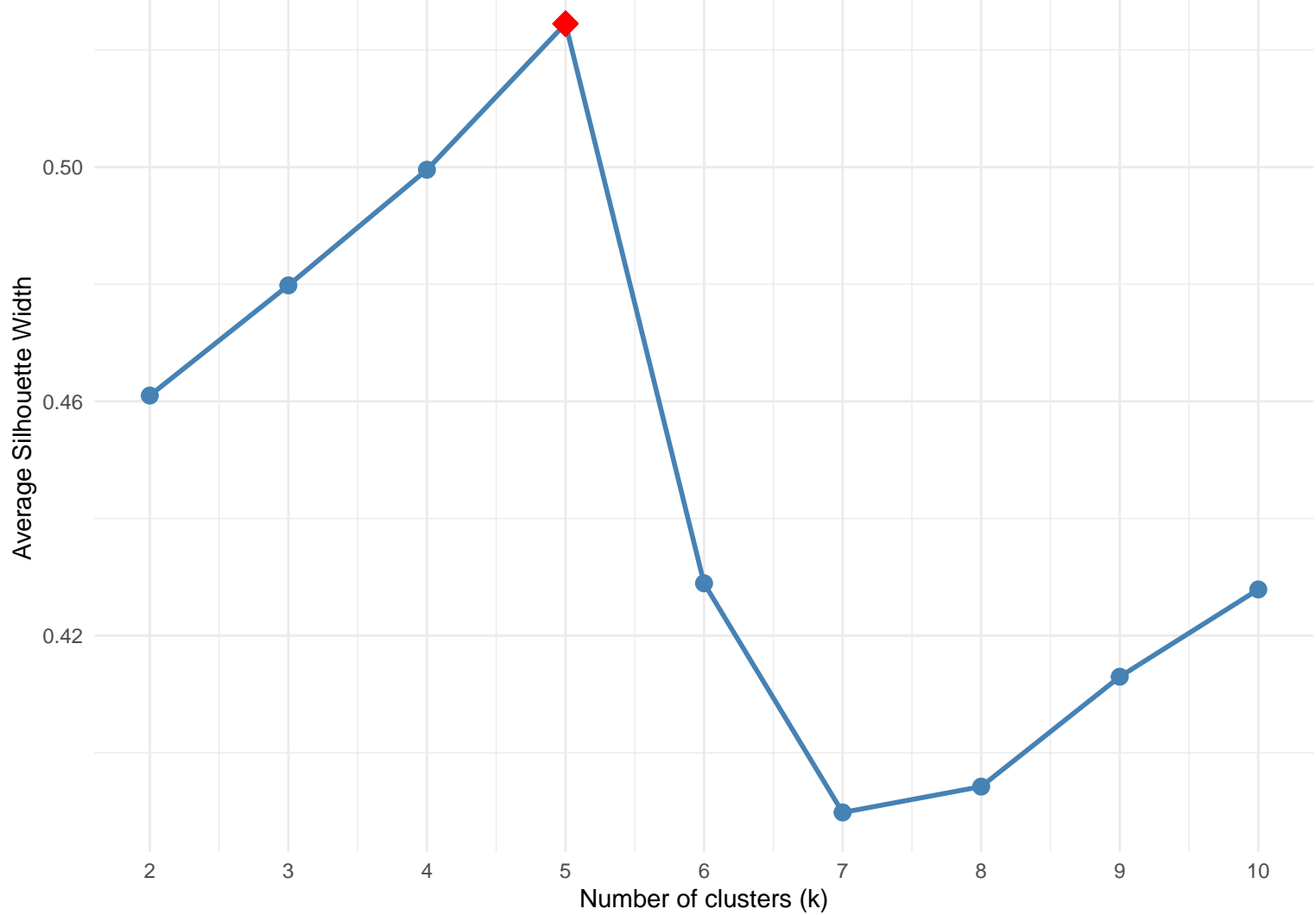

Supplement: S2 File — (Executable R script (version 4.6.0) implementing all steps: reading data, computing least-cost path distances, determining optimal k, performing k-medoids clustering, and exporting results. The script uses the raster, gdistance, sf, cluster, and clue packages.). (ZIP) [file pone.0351644.s002.zip › S2_Code/silhouette_plot_Yangshao.pdf]

# Clustering Stability Test (Jaccard Similarity)

20 random initializations vs. benchmark (seed = 42)

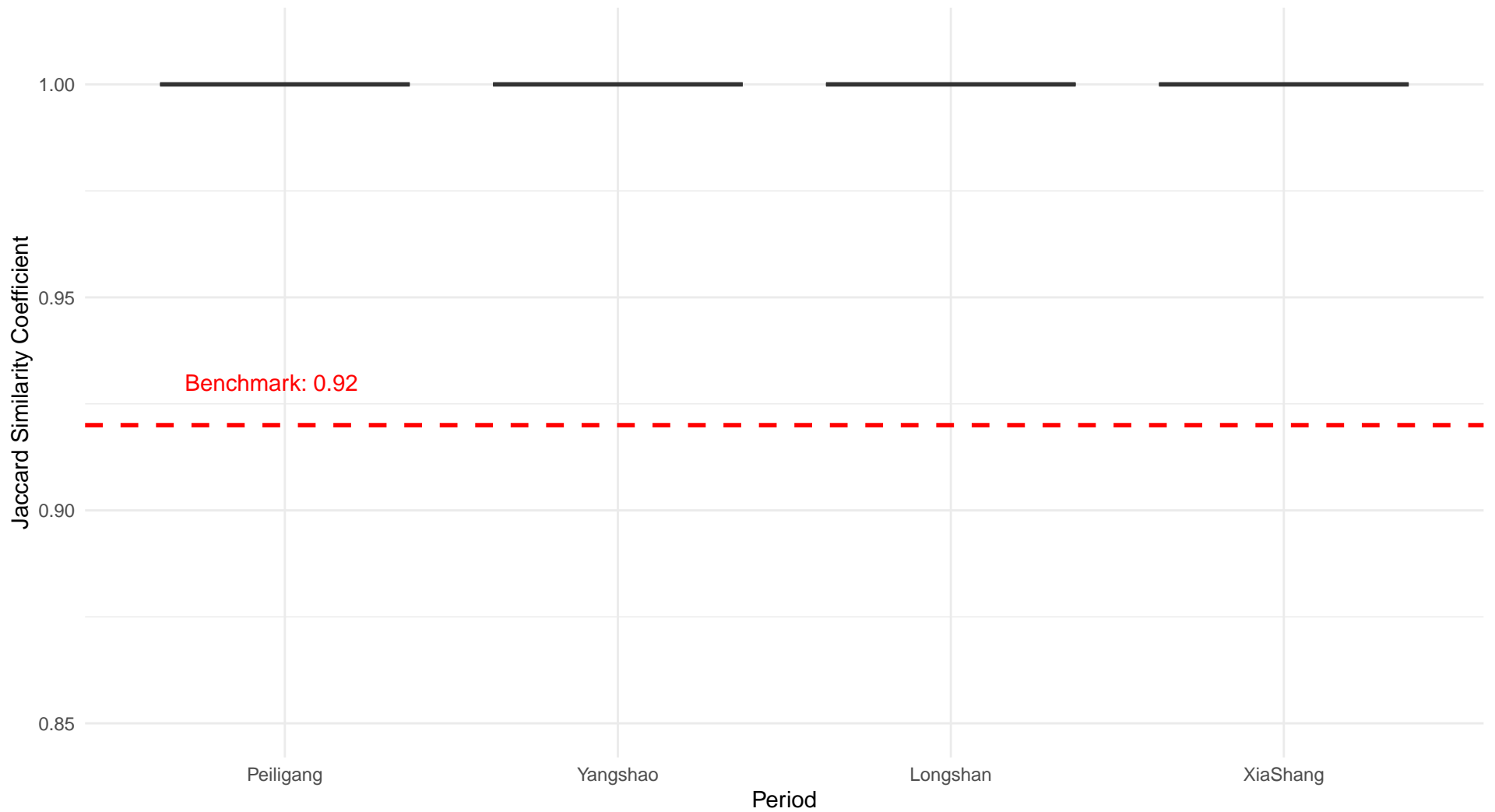

Supplement: S2 File — (Executable R script (version 4.6.0) implementing all steps: reading data, computing least-cost path distances, determining optimal k, performing k-medoids clustering, and exporting results. The script uses the raster, gdistance, sf, cluster, and clue packages.). (ZIP) [file pone.0351644.s002.zip › S2_Code/stability_plot.pdf]
